# A 10-year retrospective analysis of sudden unexpected death in the young investigated at Salt River Mortuary, Cape Town

**DOI:** 10.1007/s00414-024-03331-y

**Published:** 2024-09-17

**Authors:** Micaela Louise Swart, Yuvika Vandayar, Calvin Gerald Mole, Ogheneochuko Oghenechovwen, Dirk Hamadziripi, Laura Jane Heathfield

**Affiliations:** Division of Forensic Medicine and Toxicology, Department of Pathology, Faculty of Health Sciences, University of Cape Town, Anzio Road, P.O. Box 13914, Observatory, 7925 South Africa

**Keywords:** SUDY, Sudden death, Risk factors, Salt River Mortuary, South Africa

## Abstract

Sudden unexpected death in the young (SUDY) is defined as the rapid, unsuspected demise of an apparently healthy individual between the ages of one and 40 years. There is a gap in research pertaining to this population in a South African context. This retrospective study aimed to explore the burden, scope of post-mortem investigation, and risk factors of SUDY admissions to Salt River Mortuary (SRM) in Cape Town between 1 January 2010 and 31 December 2019. Medico-legal case files pertaining to SUDY cases from SRM were reviewed. SRM received a total of 34 601 admissions in the 10-year period; of which 1 997 (5.77%) were SUDY cases. Nearly two-thirds (62.59%) of the SUDY admissions were male. The leading cause of death was pneumonia (17.11%), and the most prevalent organ system implicated in cause of death was the pulmonary system (45.19%). At least 32.46% of SUDY cases were infectious-related, with varying degrees of confidence. A large proportion of cases had no history of acute or chronic illness (45.43%), and no family history of illness (56.66%). In total, 52 potential candidates were identified for a molecular autopsy, of which 47 have stored biological samples for future investigations. This study advocates for the routine performance of post-mortem ancillary microbiological and toxicological testing in cases of SUD, considering the large burden of infectious disease and substance abuse in South Africa. The retention of biological samples in undetermined or non-specific natural cases is also urged, to allow for cause of death determination on a molecular level.

## Introduction

Sudden unexpected death in the young (SUDY) is characterised by a rapid and unanticipated episode that results in the death of a seemingly healthy individual aged between one and 40 years [1]. SUDY is thought to affect approximately 1–3 per 100 000 young people annually [2–4], however, the incidence of SUDY in African countries is largely unknown [5]. Investigating cause of death, demographics, physical profile, as well as lifestyle and medical history pertaining to SUDY has previously assisted in identifying risk factors, which enables the development of intervention strategies to mitigate such cases in the future [5].

In most countries, deaths that are sudden and unexplained are referred for medico-legal investigations to determine the ultimate cause of death, which may include a death scene investigation, autopsy, and a review of medical and social history. Cause of death for these cases is often reported to be cardiac or epileptic in nature [5–8], although a study conducted in Pretoria, South Africa found that the leading cause of death in SUD in children (SUDC) was of pulmonary origin. Cause of death in SUDY thus may be linked with age as well as geographical location [4, 7, 9–11].

Cause of death may be diagnosed at autopsy or with ancillary investigations [11, 12]. However, some cases present with no macroscopic or microscopic pathologies, and the cause of death remains undetermined [13, 14]. These cases are termed ‘autopsy-negative’, and such outcomes may have a profound effect on the family and their ability to process and accept the death of a loved one [3]. In South Africa, an ‘undetermined’ cause of death is a final categorisation which indicates uncertainty despite all efforts to determine the cause of death, while ‘under investigation’ is a temporary categorisation allocated to cases where cause of death is not known, however, the post-mortem investigation into cause of death determination is still in progress. Up to one third of autopsy-negative cases may be attributable to cardiac channelopathies, which can only be diagnosed through DNA testing [15]. It is possible that the findings of a molecular autopsy could explain at least a portion of unexplained cases, which would bring some level of closure to relatives [16–19].

Salt River Mortuary (SRM) (recently replaced by the Observatory Forensic Pathology Institute) is an academic mortuary that services the western metropole of the City of Cape Town in South Africa. The mortuary has a historically large caseload [20], with 4 500 unnatural death investigations in 2023 alone (in-house data). An assessment of SUDY cases has not previously been conducted in this region, and information regarding the frequency and profile of these cases, the presence of known risk factors, the scope of post-mortem investigations and the ultimate causes of death are currently unknown. The aim of this study was therefore to retrospectively explore these factors in SUDY admissions to SRM over a ten-year period (1 January 2010 – 31 December 2019).

## Methodology

### Study population and case identification

The autopsy records from all admissions to SRM that occurred between 2010 and 2019 (*n* = 34 601) were reviewed using the Office Autopsy Database (HREC: R036/2014) to identify SUDY cases. The inclusion criteria were cases that were admitted to SRM between 1 January 2010 and 31 December 2019 (inclusive) as ‘sudden unexpected death’, with the age at death ranging between 365 days and 40 years. Cases involving partial or skeletonised human remains, were excluded as such cases could not be accurately attributed to the definition of sudden and unexpected death (SUD). Decomposed bodies were included as they met the inclusion criteria, but it is acknowledged that these cases may have skewed body mass index (BMI) data. Molecular autopsy candidates were selected based on the following criteria:

- SUDY cases admitted to SRM between 2010 and 2019,
- ‘Undetermined’, ‘under-investigation’ or ‘non-specific/semi-specific natural’ cause of death following a full post-mortem investigation with ancillary investigations,
- Clinical history of heart disease and/or family history of heart disease and/or sudden death.

### Data collection

After applying the inclusion and exclusion criteria, 1 997 cases were identified, and medico-legal case files were retrieved. Variables pertaining to demographics, circumstances at death, known risk factors, details of post-mortem investigation, as well as medical and social histories were collected into Microsoft Excel (Table 1).

**Table 1:** Variables collected from the SUDY medico-legal case files

Categories collected	Variables	Availability of data
*Demographics and physical profile*	Age Sex Body mass index (BMI)	1 997/1 997 (100.00%) 1 917/1 997 (95.99%) 1 791/1 997 (89.68%)
*Other Profile*	Geographical location of death Location of death Position at death Activity at death Season of death	1 997/1 997 (100.00%) 1 979/1 997 (99.10%) 1 262/1 997 (63.19%) 936/1 997 (46.87%) 1 997/1 997 (100.00%)
*Extent of post-mortem*	Full, partial, external only Ancillary investigations Cause of death Manner of death Organ system implicated in death	1 930/1 997 (96.64%) 1 931/1 997 (96.70%) 1 932/1 997 (96.75%) 1 932/1 997 (96.75%) 1 217/1 460 (83.36%)
*Medical history*	Symptoms prior to death Acute/chronic illness(es) during lifetime Medication(s) taken Family medical history	1 454/1 831 (79.41%) 1 594/1 831 (87.06%) 1 430/1 831 (78.10%) 909/1 831 (49.65%)
*Social history*	Highest level of education Annual household income Type of housing Marital status Substance(s) used during lifetime Occupation	137/287 (47.74%) 37/287 (12.89%) 1 015/1 831 (55.43%) 1 593/1 831 (87.00%) 1 474/1 831 (80.50%) 1 565/1 831 (85.47%)

BMI was calculated using the conventional equation BMI = mass (kg)/height (m)^2^. For decedents < 18 years old, BMI was interpreted in relation to age and sex, based on the World Health Organization BMI-for-age-and-sex standards [21, 22]. For decedents ≥ 18 years old, a BMI < 18.5 was recorded as ‘underweight’, and a BMI > 24.9 was recorded as ‘overweight’. A BMI within the range of 18.5–24.9 was recorded as ‘normal’.

Socioeconomic factors were obtained from routine questionnaires of next-of-kin during the post-mortem investigation, which are available in the case files. The effect of socioeconomic factors such as marital status has been associated with health and mortality in the literature [23, 24]. Marital status of adults was recorded where available. For minors (< 18 years of age), marital status of the minor’s mother/guardian was recorded. Highest level of education and annual household income were collected for minors, as details pertaining to these variables are routinely requested for these cases.

Double entry validation was carried out on all included cases. The 10-year dataset was then cleaned to ensure consistency and uniformity before data analysis.

### Data analysis

Descriptive statistics were carried out to summarise the data. Numerical data were assessed with the Shapiro-Wilk test for normality. Differences between numerical data were assessed using Student’s t-tests for normally distributed data and Wilcoxon rank-sum tests where data did not conform to normality. Associations between categorical data were assessed using Pearson’s Chi-squared test. Where assumptions of this test were violated Fisher’s Exact test was applied. Pairwise differences between proportions within categorical variables were assessed post-hoc with Bonferroni correction applied. A p-value of ≤ 0.05 was deemed statistically significant for all tests. Statistical analyses were performed using the Statistical Package for Social Sciences (SPSS) Ver. 24 (IBM corp., Armonk, NY).

Notably, details pertaining to *every* variable in this study were not always available for every case. This was also noted by Tiemensma et al. [12], and may be either due to missing documents, absence of a response, language barriers, or the details were not specified. As a result, the denominator for each variable is different, reflecting the variability in availability of data per variable.

## Results

### Study population

**Fig. 1:**
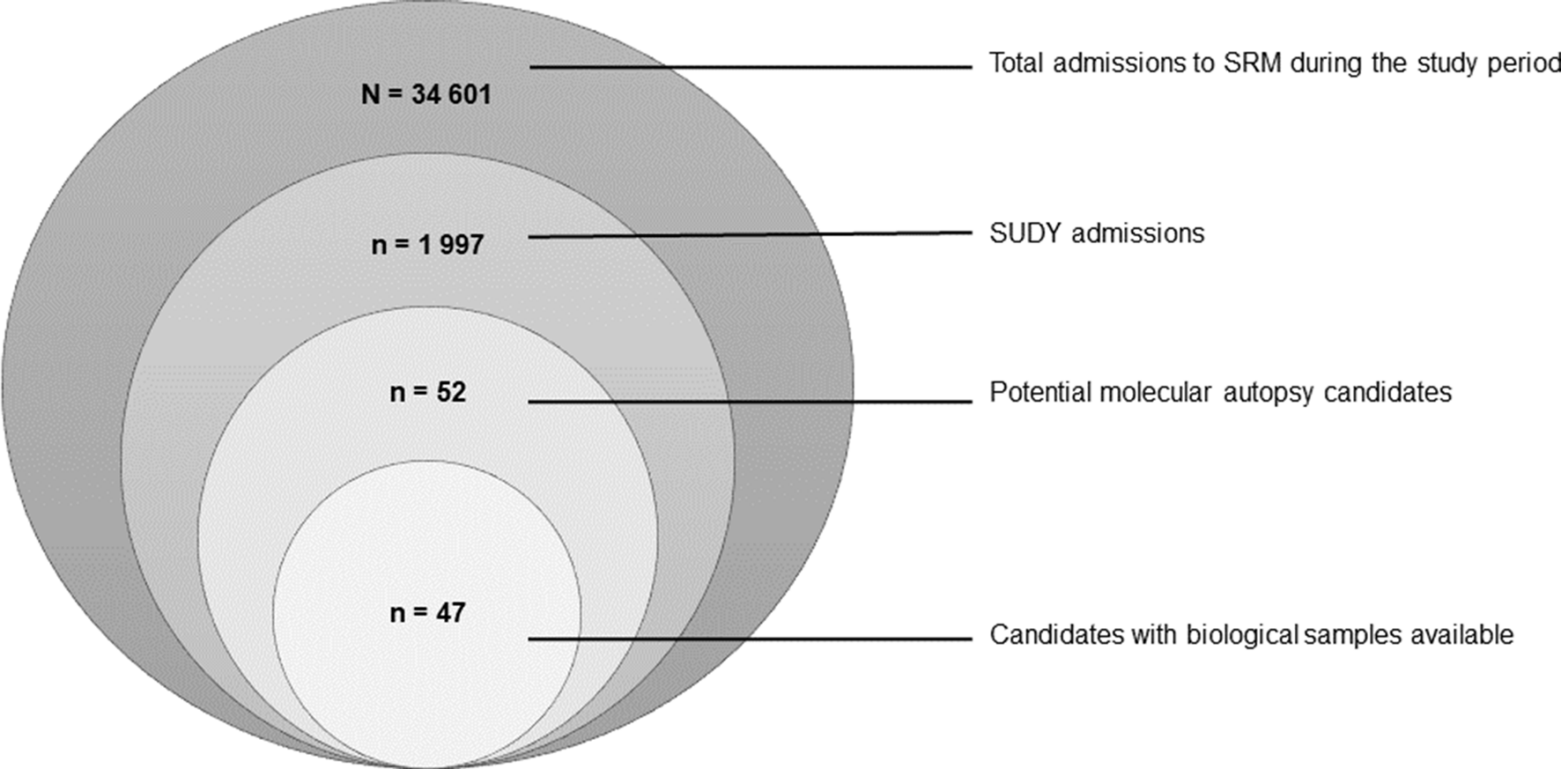
Onion chart representing the total number of cases admitted to SRM between 1 January 2010 and 31 December 2019, and the subdivisions of cases relevant to the study

SRM received a total of 34 601 admissions between 1 January 2010 and 31 December 2019. The total number of SUDY admissions to SRM in the 10-year study period was 1 997, thus comprising 5.77% of the 10-year caseload. This equates to approximately 200 SUDY admissions to SRM annually. A total of 52 cases were identified as potential candidates for a molecular autopsy, and of these, 47/52 (90.38%) had biological samples available for future investigations (Fig. 1).

### Demographics and physical profile of the SUDY cases

#### Age and sex

The mean age for the overall SUDY population was 26.89 ± 10.87 years. The mean age of the male and female SUDY admissions was 27.21 ± 10.89 and 26.35 ± 10.82 years, respectively. Within the children age category, there was a significantly greater proportion of females compared to males (*p* = 0.028). No significant difference between the proportion of male and female was observed in the toddler, teenager or adult age categories.

Males accounted for the majority (1 250/1 997; 62.59%) of the SUDY admissions within the 10-year study period. However, the male SUDY admissions represented only 4.7% (1 250/26 596) of the total male admissions to SRM over the 10 years, whereas the female SUDY admissions represented 9.43% (747/7 925) of the total female admissions to SRM over the 10 years. There were 80 admissions to SRM over the study period with undetermined sex. Upon dividing the study population into SUDC (1–18 years) and SUD in adults (SUDA) (18–40 years) age groups, these groups accounted for 0.90% and 4.87% of the 10-year SRM caseload, respectively.

**Fig. 2:**
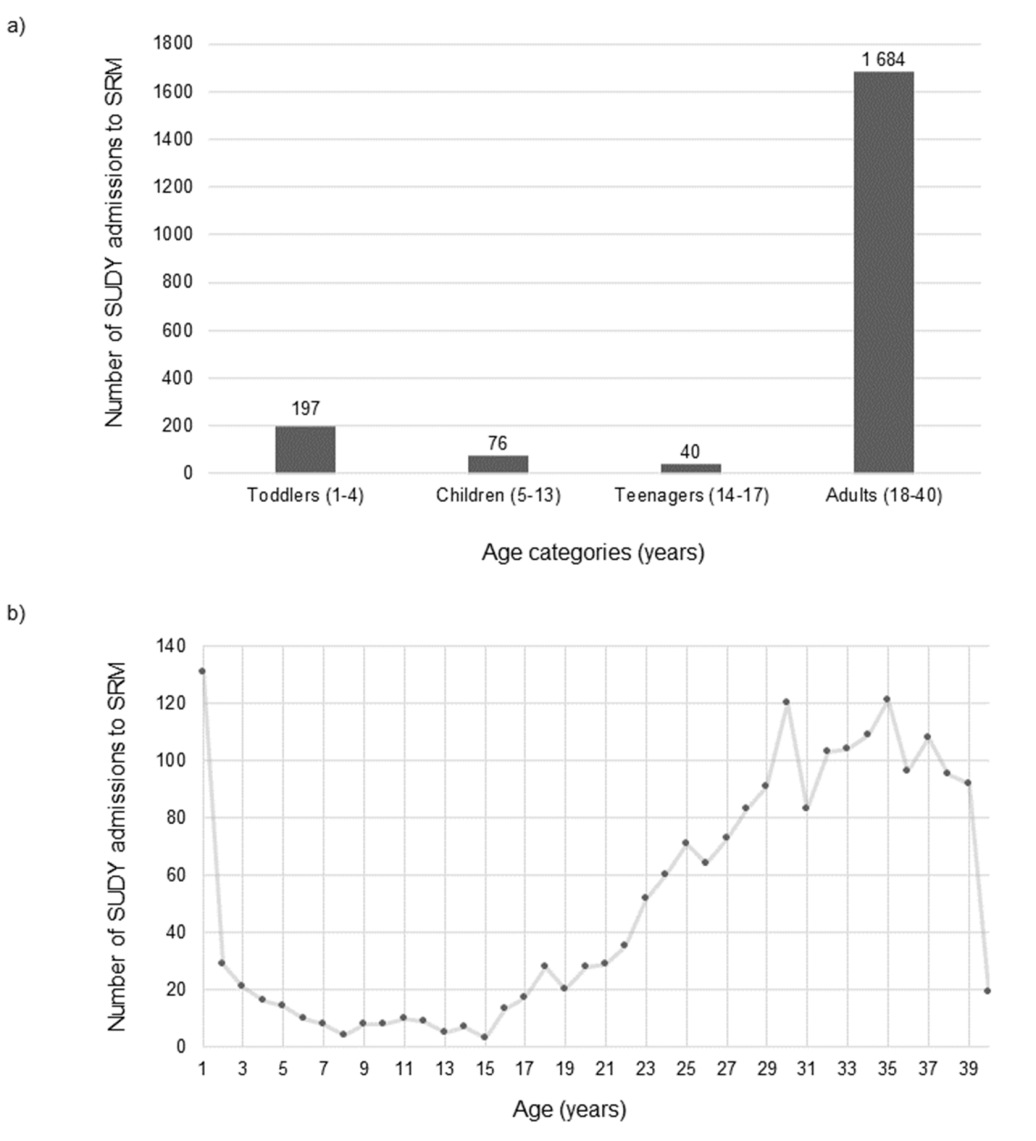
The number of SUDY admissions to SRM by (**a**) age categories and (**b**) individual age across the study period 2010–2019

With respect to the prevalence of admissions across the ages, from 1 to 40 years, one-year olds comprised the largest individual age group of SUDY admissions to SRM (6.56%), and 67.01% (132/197) of SUDY admissions within the 1–4 year age category (Fig. 2). SRM received few 2–15 year old SUDY admissions. More SUDY cases were received from older age groups, peaking again between the ages 30–35 years. The 1–4 year old SUDY admissions comprised the largest proportion of SRM admissions per age category for the study period (197/603; 32.67%), based on the Office Autopsy Database.

#### BMI

**Fig. 3:**
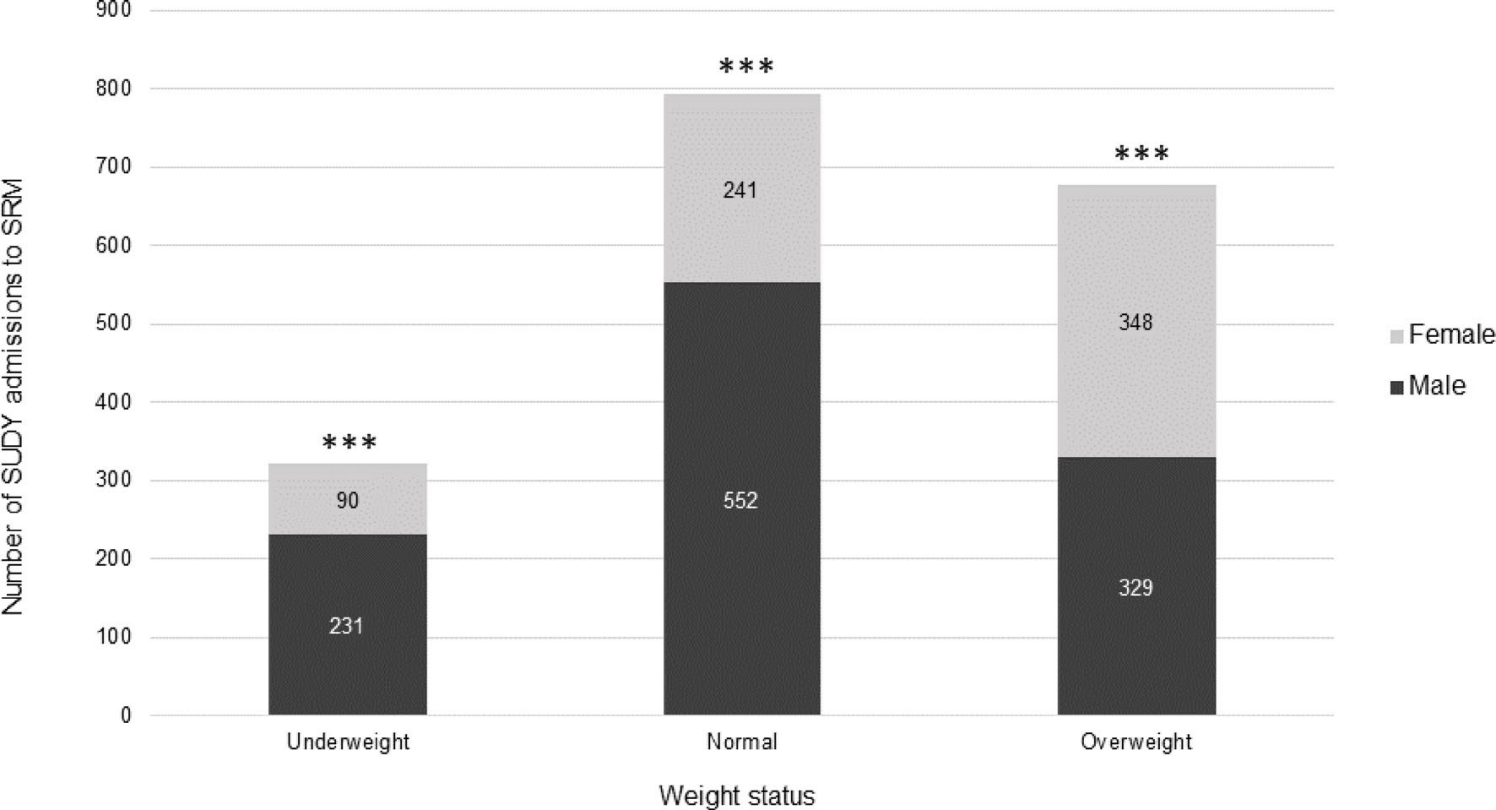
The weight status of the SUDY admissions to SRM, and the sex composition of each weight status. * Indicates significant difference between sexes in each weight status category

The mean BMI of the SUDY admissions to SRM in the 10-year period was 23.44 ± 7.22. The ‘underweight’ group may be overrepresented as several SUDY admissions comprised decomposed remains. The BMI and weight status is presented in Fig. 3 for males and females. The mean female and male BMI were 25.73 ± 7.22 and 22.04 ± 7.22 (*p* < 0.01), respectively. A significant association was identified between sex and weight status (*p* < 0.001). The male sex accounted for the majority of the underweight (*p* < 0.001) and normal admissions (*p* < 0.001), whereas the female sex accounted for majority of the overweight admissions (*p* < 0.001).

The pulmonary system (*n* = 552; 30.16%) and cardiovascular system (*n* = 392; 21.42%) were the two most prominent organ systems associated with death in SUDY cases. Weight status was significantly associated with involvement of the cardiovascular system (*p* < 0.001) with a significantly greater proportion of individuals with cardiovascular involvement being classified as overweight (*p* < 0.001). Similarly, weight status was significantly associated with involvement of the pulmonary system (*p* < 0.001), however a significantly greater proportion of individuals with pulmonary involvement were classified as underweight (*p* < 0.001).

### Profile of the SUDY admissions to SRM

#### Geographical location of the SUDY admissions

All incidents of SUD are to be reported to the nearest South African Police Services (SAPS) station [25]. The SAPS station that received the highest number of SUDY cases in the 10-year study period was Nyanga (294/1 997; 14.72%), followed by Gugulethu (184/1 997; 9.21%) and Mitchell’s Plain (178/1 997; 8.91%).

#### Monthly and seasonal distribution of SUDY admissions to SRM

**Fig. 4:**
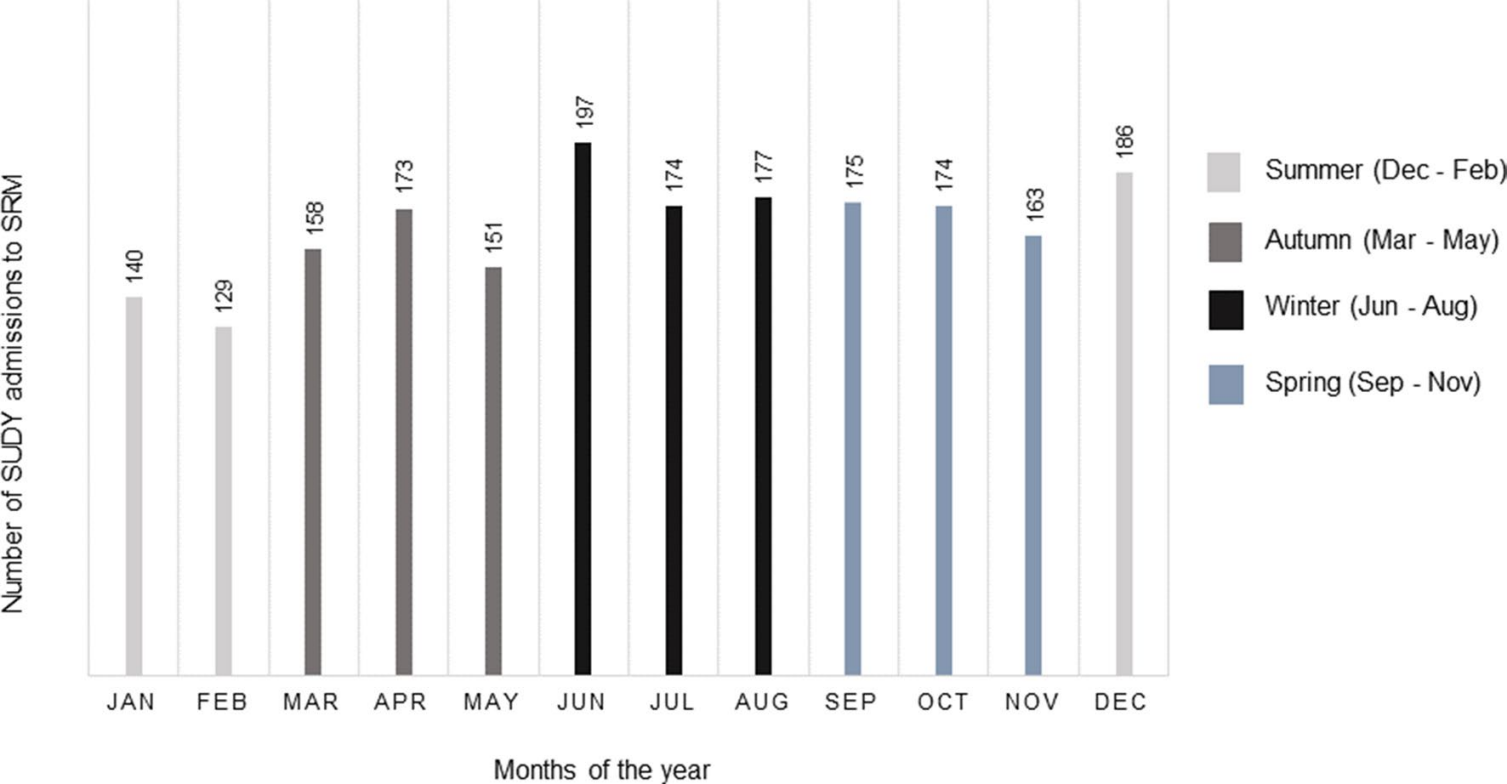
The cumulative monthly and seasonal distribution of SUDY admissions to SRM during the 10-year study period

The cumulative monthly SUDY admissions to SRM in the 10-year study period peaked in June and December (Fig. 4), representing a seasonal peak at the beginning of winter and summer, respectively, in a South African context. The winter season experienced the highest frequency of SUDY, followed by spring, autumn, and summer (*p* = 0.0233).

#### Circumstances surrounding death

**Fig. 5:**
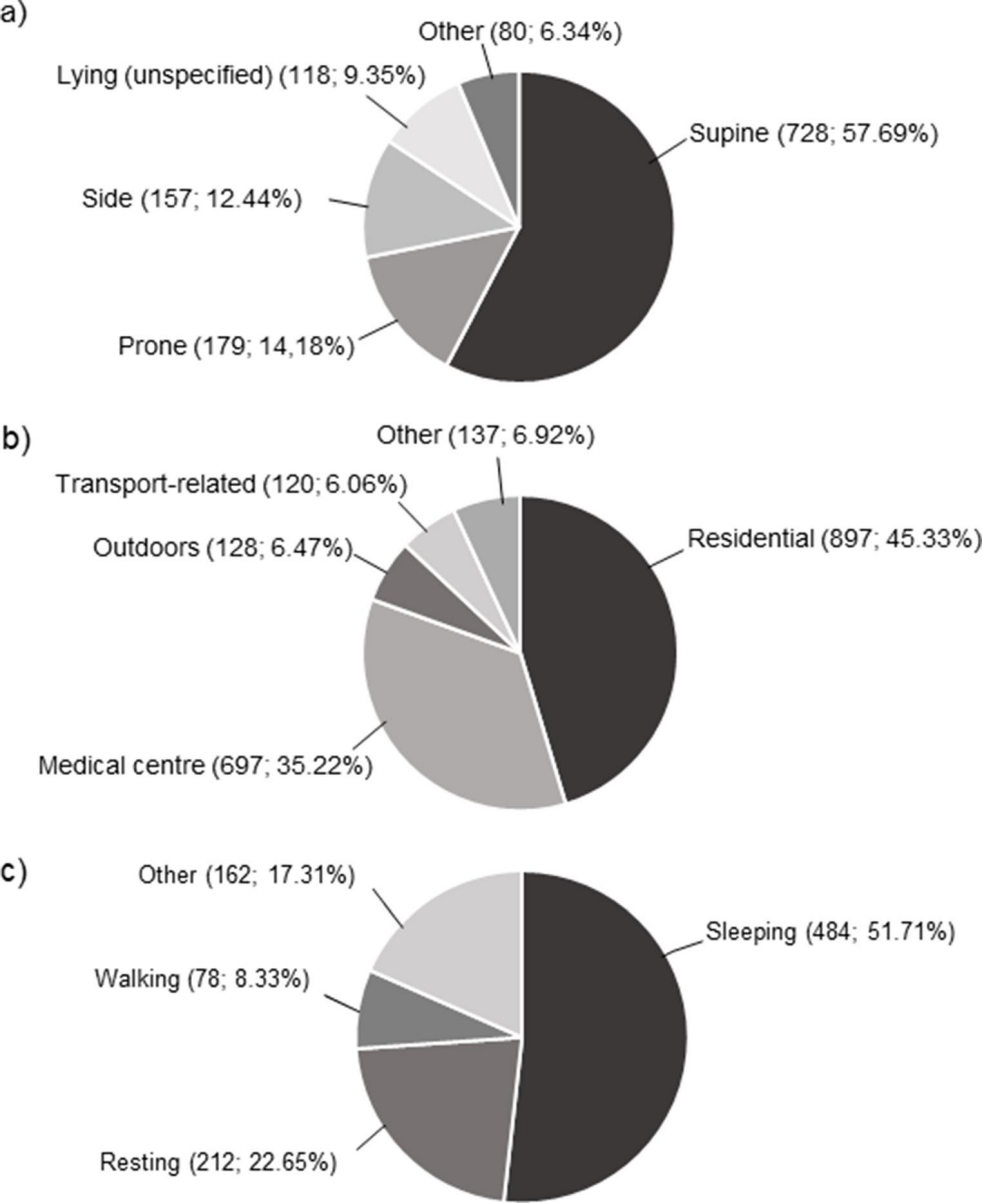
Pie charts depicting the (**a**) position, (**b**) location, and (**c**) activity at/before death of the SUDY admissions to SRM during the 10-year study period

Where specified, the most frequently recorded position at death was ‘lying down’, more specifically supine (728/1 262; 57.69%), prone (179/1 262; 14.18%), and side (157/1 262; 12,44%) (Fig. 5a). A significant association was identified between sex and position at death (*p* = 0.002). A significantly greater proportion of males were found lying prone (*p* < 0.001) or supine (*p* = 0.0251) at death than females. No other positions were significantly associated with sex. Position at death was significantly associated with age group (*p* < 0.001), with toddlers having a greater risk of dying in a supine position or lying on their side than other age groups.

Where specified, the most frequently reported locations at death were residential (897/1 979; 45.33%) and medical centre (697/1 979; 35.22%) (Fig. 5b). A significant association was identified between age and location at death (*p* = 0.001), with more adults dying outdoors (*p* < 0.001), and in residential settings (*p* = 0.0214). Toddlers had a greater risk of being declared dead at a medical centre (*p* < 0.001). Sex was significantly associated with location at death (*p* < 0.001). A significantly greater proportion of males were found outdoors (*p* < 0.001), with females having a greater risk of being declared dead at medical centres (*p* < 0.001).

Where specified, the most frequently reported activity at/before death was ‘sleeping’ (484/936; 51.71%), and ‘resting’ (212/936; 22.65%) (Fig. 5c). A significant association was identified between age and activity at/before death (*p* < 0.001), with more toddlers dying while eating, and more children and toddlers dying while at play or sleeping. A significant association between sex and activity at/before death was observed (*p* < 0.001). A greater proportion of males died during physical activity, while a greater proportion of females died during domestic work.

### Extent of post-mortem investigation and cause of death

Following a post-mortem investigation, the cause of death was deemed natural, under investigation, unnatural and undetermined in 1 460/1 931 (75.56%), 280/1 931 (14.50%), 101/1 931 (5.23%) and 91/1 931 (4.71%) of cases, respectively. Where affidavits were available, the extent of post-mortem investigation was external in 724/1 930 (37.51%) cases, partial in 134/1 930 (6.94%) cases, and full in 1 072/1 930 (55.54%) cases.

Of the cases with a presumed manner of death, ancillary investigations were performed in 1 873/1 931 (97.00%) cases. The frequency of ancillary investigations performed is depicted in Table 2.

**Table 2:** The number of post-mortem ancillary investigations performed on the SUDY cases according to the manner of death [N (% manner of death)]

Manner of death	Ancillary investigations					
	Toxicology	Histology	Microbiology	Virology	LODOX scan	Other
Natural (*n* = 1 460)	225 (15.41%)	358 (24.52%)	122 (8.36%)	22 (1.51%)	1 363 (93.36%)	71 (4.86%)
Under investigation (*n* = 280)	261 (93.21%)	206 (73.57%)	27 (9.64%)	3 (1.07%)	265 (94.64%)	29 (10.36%)
Unnatural (*n* = 101)	68 (67.33%)	46 (45.54%)	8 (7.92%)	1 (0.99%)	94 (93.07%)	11 (10.89%)
Undetermined (*n* = 91)	70 (76.92%)	63 (69.23%)	10 (10.99%)	2 (2.20%)	84 (92.31%)	11 (12.09%)
Totals	**624 (32.31%)**	**673 (34.85%)**	**167 (8.64%)**	**28 (1.45%)**	**1 806 (93.53%)**	**122 (6.32%)**

[‘Other’ may refer to cell count, neuropathology, chemical pathology, immunology etc.]

Unnatural deaths did not undergo any further analysis as they do not meet the study criteria. Additionally, deaths with a missing cause of death outcome (65/1 997; 3.25%) were excluded to avoid misrepresentation of possible risk factors for SUDY.

The organ system involved in the cause of death of natural SUDY cases was not specified in 243/1 460 (16.64%) cases. When specified, the main organ system implicated was the pulmonary system (550/1 217; 45.19%), followed by the cardiovascular system (387/1 217; 31.80%), and central nervous system (166/1 217; 13.64%) (Table 3). The greatest overlap of organ systems existed between the pulmonary and cardiovascular systems (63/1 217; 5.18%).

**Table 3:** The two leading causes of death per organ system and age category

	Leading Causes of Death	Frequency per System
**Organ system**		
Pulmonary	1. Pneumonia 2. Pulmonary TB	137/338 (40.53%) 91/338 (26.92%)
Cardiovascular	1. Ischaemic heart disease 2. Myocardial infarction	43/225 (19.11%) 36/225 (16.00%)
Central Nervous System	1. Meningitis 2. Epilepsy	40/137 (29.20%) 24/137 (17.52%)
**Age categories (years)**		
1–4 (toddlers) 5–13 (children) 14–17 (teenagers) 18–40 (adults)	1. Pneumonia 2. Gastroenteritis 1. Pneumonia 2. Myocarditis 1. Pneumonia 2. Pulmonary TB Meningitis Epilepsy Infective endocarditis Bacterial septicaemia Acute bronchial asthma Dilated Cardiomyopathy of uncertain etiology Sudden arrhythmic event Consistent with hypovolaemic shock 1. TB 2. Pneumonia	21/83 (25.30%) 19/83 (22.89%) 8/36 (22.22%) 6/36 (16.67%) 2/11 (18.18%) 1/11 (9.09%) 1/11 (9.09%) 1/11 (9.09%) 1/11 (9.09%) 1/11 (9.09%) 1/11 (9.09%) 1/11 (9.09%) 1/11 (9.09%) 1/11 (9.09%) 113/700 (16.14%) 111/700 (15.86%)

The overall leading cause of death in specified natural cases was pneumonia (142/830; 17.11%), followed by tuberculosis (TB) (117/830; 14.10%). Previous TB-exposure during lifetime was only reported in 57/117 (48.72%) SUDY cases whose cause of death was determined to be TB. The leading causes of death in specified natural deaths were assessed by organ system and age category (Table 3).

**Fig. 6:**
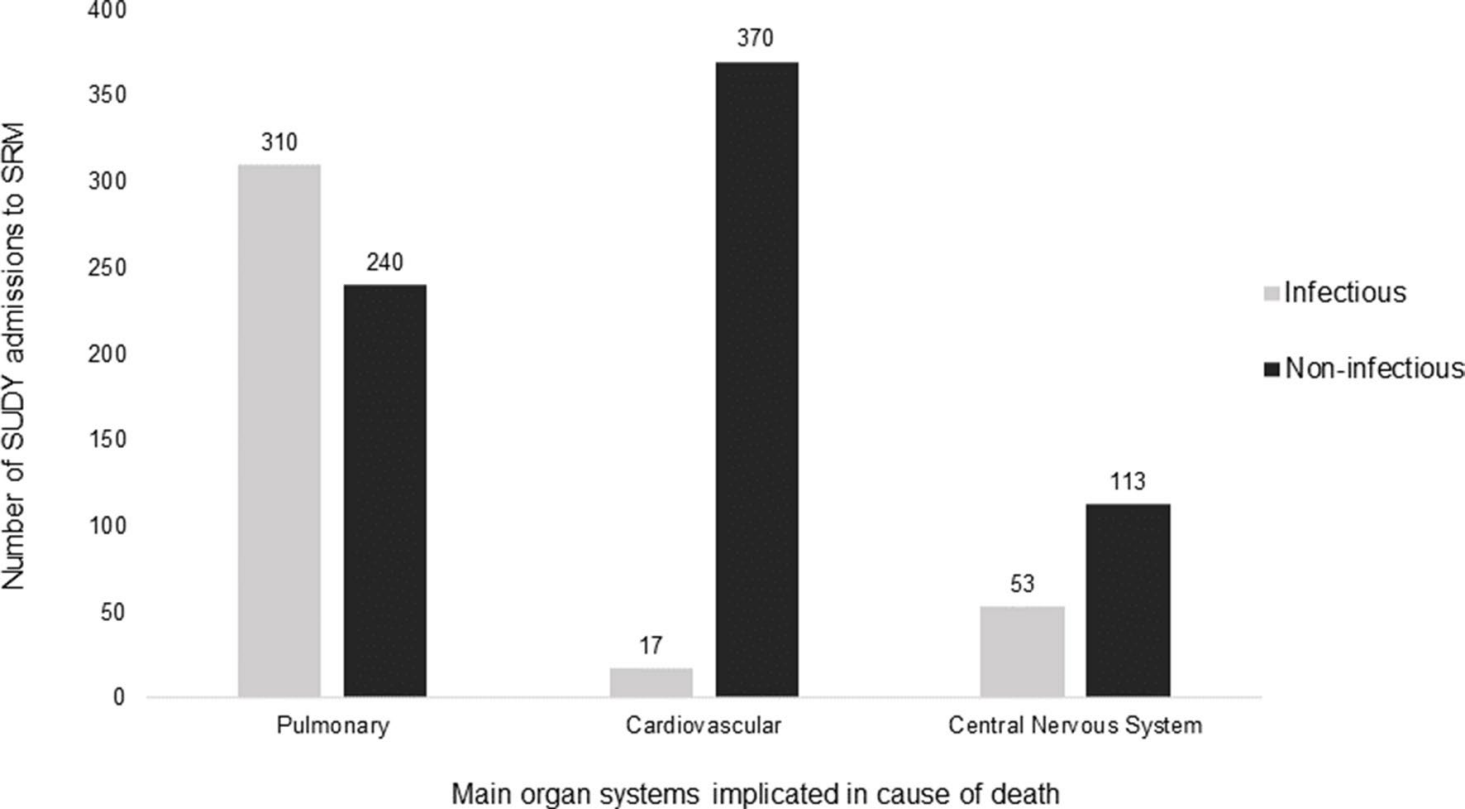
Bar graph representing the infectious and non-infectious related deaths by organ system implicated in specific and semi-specific natural causes of SUDY

Notably, infectious diseases accounted for at least 396/1 220 (32.46%) of specific/semi-specific natural causes of SUDY cases. The proportion of infectious and non-infectious related deaths pertaining to the main organ systems implicated in cause of death is depicted in Fig. 6.

### Social history

Majority of the SUDY admissions were that of unmarried individuals, or of minors (< 18 years) with unmarried mothers/guardians (1 243/1 593; 78.03%) (Table 4). Where specified, nearly all (135/137; 98.54%) mothers of minors received some level of education, of which the majority completed a secondary level of education (117/135; 86.67%). The type of housing in which the decedents lived was fairly equally distributed between formal (528/1 015; 52.02%) and informal (468/1 015; 46.11%) (Table 4). Excluding minors and students, 42.88% (671/1 565) of decedents were not economically active. The annual household income was captured for minors (< 18 years old) only and ranged from R0.00 to R780 000.00, with a mean annual income of R60 566.67.

**Table 4:** Social history of SUDY victims

	Total	Male	Female
**Marital status**			
*Married*	350/1 593 (21.97%)	200/350 (57.14%)	150/350 (42.86%)
*Unmarried*	1 243/1 593 (78.03%)	773/1 243 (62.19%)	470/1 243 (37.81%)
*Unknown*	238/1 831 (13.00%)	-	-
**Type of housing**			
*Formal*	528/1 015 (52.02%)	337/528 (63.83%)	191/528 (36.17%)
*Informal*	468/1 015 (46.11%)	299/468 (63.89%)	169/468 (36.11%)
*Prison*	18/1 015 (1.77%)	17/18 (94.44%)	1/18 (5.56%)
*Other*	1/1 015 (0.10%)	1/1 (100%)	0/1 (0%)
*Unknown*	816/1 831 (44.57%)	-	-
**Occupation**			
*Unemployed*	615/1 565 (39.30%)	341/615 (55.45%)	274/615 (44.55%)
*Employed*	576/ 1 565 (36.81%)	387/576 (67.19%)	189/576 (32.81%)
*Minor (< 18 years)*	287/1 565 (18.34%)	172/287 (59.93%)	115/287 (40.07%)
*Vagrant*	38/1 565 (2.43%)	25/38 (65.79%)	13/38 (34.21%)
*Student*	31/1 565 (1.98%)	17/31 (54.84%)	14/31 (45.16%)
*Imprisoned*	18/1 565 (1.15%)	17/18 (94.44%)	1/18 (5.56%)
*Unknown*	266/1 831 (14.53%)	-	-

**Fig. 7:**
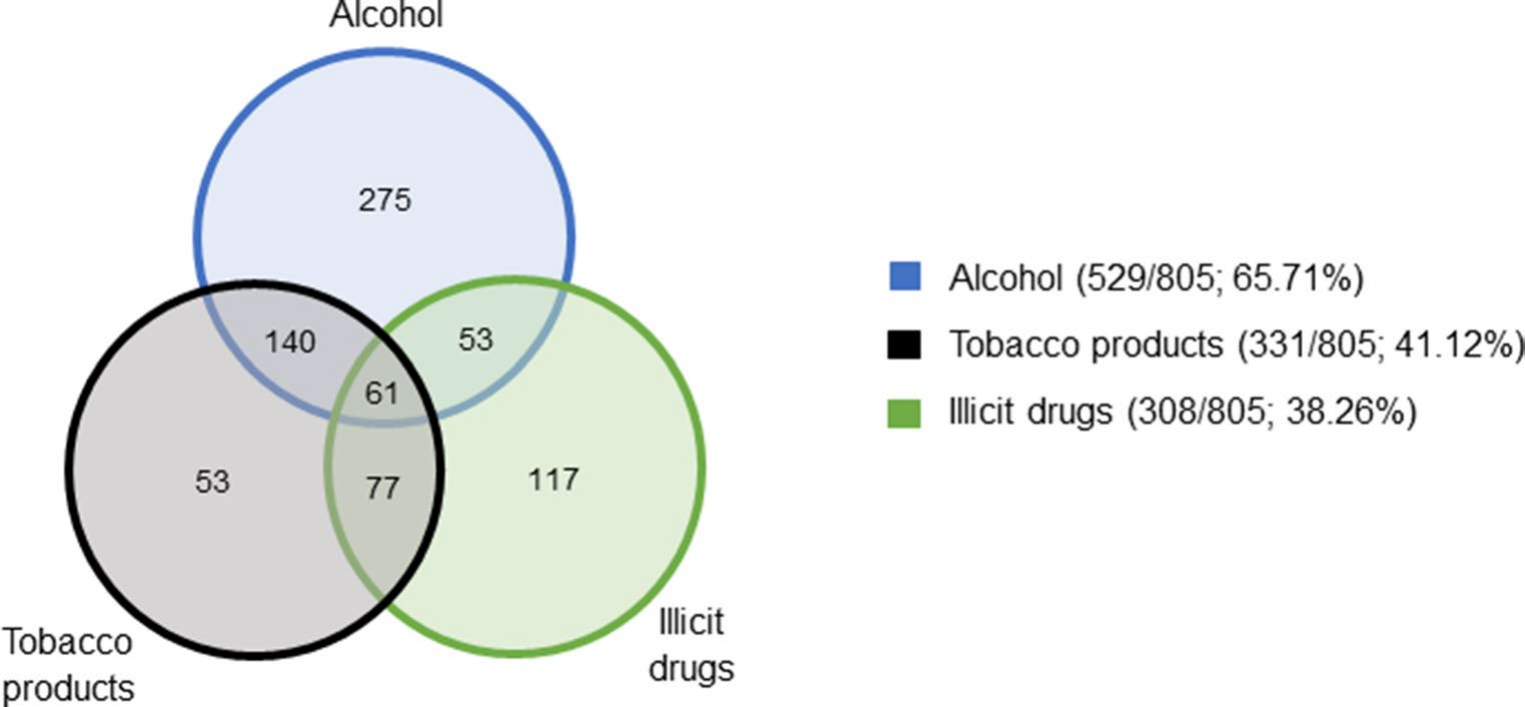
Venn diagram depicting substance use among SUDY admissions to SRM over 10 years

Where available (1 474/1 831; 80.50%), 805/1 474 (54.61%) SUDY cases reported that some form of substance, either alcohol, tobacco products, or illicit drugs, or a combination, was engaged with during the deceased’s lifetime (Fig. 7). Of these, 29/805 (3.60%) cases did not specify which substance. Underaged substance-use was noted in seven cases, of which 3/7 engaged with tobacco products only, 2/7 engaged with alcohol only, 1/7 engaged with illicit drugs only, and 1/7 engaged with a combination of alcohol and illicit drugs.

A total of 42 individuals were reported to have engaged in physical activity. The leading organ system implicated in the cause of death of these individuals was the cardiovascular system (21/42; 50%).

### Medical history

The medical history of the SUDY victims was collected with the aim of identifying various symptoms, chronic or acute illnesses, or family history that may place one at

risk for SUD. Chronic/acute illnesses or infections, and family history that contributed to > 5% of SUDY cases are depicted.

Symptoms prior to death were unknown/not reported in 377/1 831 (20.59%) cases. Where specified, symptoms preceding death were experienced in at least 1 363/1 454 (93.74%) cases (Table 5). The leading symptom prior to death was breathlessness, experienced in almost a third of cases (450/1 363; 33.02%). ‘Breathlessness’ was the predominant symptom experienced in the age categories 5–13 and 18–40 years and in both sexes, while ‘vomiting (no blood)’ and ‘fitting’ predominated in the 1–4 and 14–17 year age categories, respectively. ‘Vomiting’ and ‘coughing’ were divided into ‘(no blood)’ and ‘blood’ categories, as the aetiology of these symptoms differ.

**Table 5:** The leading symptoms experienced by SUDY victims prior to death per age category [N (% age category)] and sex [N (% sex)]

Symptoms	Total frequency	Age category distribution (years)				Sex distribution	
		1–4 (*n* = 162)	5–13 (*n* = 62)	14–17 (*n* = 31)	18–40 (*n* = 1 108)	Male (*n* = 797)	Female (*n* = 566)
*Breathlessness*	450/1 363 (33.02%)	41 (25.31%)	21 (33.87%)	9 (29.03%)	379 (34.21%)	244 (30.61%)	206 (36.40%)
*Vomiting* *(no blood)*	392/1 363 (28.76%)	86 (53.09%)	20 (32.26%)	8 (25.81%)	278 (25.10%)	214 (26.85%)	178 (31.45%)
*Chest pain*	255/1 363 (18.74%)	3 (1.85%)	5 (8.06%)	4 (12.90%)	243 (21.93%)	163 (20.45%)	92 (16.25%)
*Foaming at mouth/nose*	238/1 363 (17.49%)	10 (6.17%)	16 (25.81%)	7 (22.58%)	205 (18.50%)	133 (16.69%)	105 (18.55%)
*Abdominal pain*	238/1 363 (17.27%)	14 (8.64%)	12 (19.35%)	5 (16.13%)	207 (18.68%)	130 (16.31%)	108 (19.08%)
*Fitting*	227/1 363 (16.65%)	25 (15.43%)	15 (24.19%)	13 (41.94%)	174 (15.70%)	145 (18.19%)	82 (14.49%)
*Headache*	205/1 363 (14.99%)	6 (3.70%)	10 (16.13%)	4 (12.90%)	185 (16.70%)	96 (12.05%)	109 (19.26%)
*Fever*	154/1 363 (11.32%)	68 (41.98%)	7 (11.29%)	3 (9.68%)	76 (6.86%)	89 (11.17%)	65 (11.48%)
*Coughing* *(no blood)*	154/1 363 (11.30%)	51 (31.48%)	8 (12.90%)	0	95 (8.57%)	91 (11.42%)	63 (11.13%)
*Diarrhoea*	150/1 363 (11.02%)	64 (39.51%)	8 (12.90%)	4 (12.90%)	74 (6.68%)	83 (10.41%)	67 (11.84%)
*Vomiting* *(blood)*	87/1 363 (6.38%)	2 (1.23%)	1 (1.61%)	2 (6.45%)	82 (7.40%)	62 (7.78%)	25 (4.42%)
*Weakness*	49/1 363 (3.60%)	6 (3.70%)	9 (14.52%)	0	34 (3.07%)	22 (2.76%)	27 (4.77%)
*Paralysis*	49/1 363 (3.60%)	1 (0.62%)	8 (12.90%)	3 (9.68%)	37 (3.34%)	21 (2.63%)	28 (4.95%)
*Listlessness*	33/1 363 (2.42%)	31 (19.14%)	2 (3.23%)	0	0	17 (2.13%)	16 (2.83%)

% indicates frequency of symptom experienced per age category and per sex

Significant associations between age and symptoms were observed. Adults had a greater risk of chest pains (*p* < 0.001), teenagers had a greater risk of fitting prior to death (*p* < 0.001), and children had a greater risk of foaming at the mouth/nose (*p* = 0.001), paralysis (*p* < 0.001) and weakness before death (*p* < 0.001). In general, a greater proportion of toddlers experienced symptoms prior to death than other age groups. Significantly more toddlers had reported diarrhoea (*p* < 0.001), vomiting (no blood), coughing (no blood) (*p* < 0.001), fever (*p* < 0.001), and general listlessness (*p* < 0.001). Sex was significantly associated with symptoms with a significantly greater proportion of females having a history of breathlessness (*p* < 0.001) abdominal pain (*p* = 0.01), vomiting (no blood) (*p* < 0.001), foaming at the mouth/nose (*p* = 0.032), headache (*p* < 0.001), paralysis (*p* = 0.005), or weakness (*p* = 0.011).

At least 874/1 594 (54.83%) cases herein had a chronic or acute illness during their lifetime (Table 6). The three main chronic or acute illnesses identified were TB-exposure (210/874; 24.03%), human immunodeficiency virus (HIV)-exposure (141/872; 16.13%) and epilepsy (139/872; 15.90%). At least 394/909 (43.34%) cases herein had a family history of disease or illness, of which asthma (147/394; 37.31%) and heart disease (119/394; 30.20%) predominated.

**Table 6:** Medical history of SUDY victims

Medical history	Total frequency
**Chronic/acute illness or infection experienced during lifetime**	
TB-exposure	210/874 (24.03%)
HIV-exposure	141/874 (16.13%)
Epilepsy	139/874 (15.90%)
Hypertension	115/874 (13.16%)
Asthma	110/874 (12.59%)
Mental disease	73/874 (8.35%)
Heart condition/problem	61/874 (6.98%)
None	720/1 594 (45.43%)
Unknown	237/1 831 (12.94%)
**Family history of illness or sudden death**	
Asthma	147/394 (37.31%)
Heart disease	119/394 (30.20%)
Cancer	116/394 (29.44%)
Sudden death	107/394 (27.16%)
Hypertension	41/394 (10.41%)
Diabetes	33/394 (8.38%)
None	515/909 (56.66%)
Unknown	922/1 831 (50.35%)
**Medication taken**	
None	748/ 1 430 (52.31%)
Yes	682/1 430 (47.69%)
Unknown	401/1831 (21.90%)

## Discussion

This is the first study to investigate and document the profile and risk factors of SUDY victims in a South African setting. International research has been conducted on this population, and other forensic mortuaries in South Africa have investigated the SUDA (≥ 18 years) and SUDC (1–18 years) populations, respectively [11, 12]. Although both populations overlap with SUDY, it is critical to investigate this markedly understudied population as a whole. Considering the novel nature of this study, the findings herein cannot be fully compared in a South African context. Nevertheless, this study adds to the growing body of literature on SUD.

### Incidence of SUDY admitted to SRM

SUDY accounted for 5.77% of the 10-year caseload at SRM (Fig. 1). This is similar to a report from Australia over a 9-year study period [26]. Although the proportion of these deaths may appear small, the sudden and unexpected nature of these deaths can have an immense impact on loved ones. It is also probable that the proportion of these deaths may be understated as a result of the large homicide burden experienced by SRM [27].

Two local studies found that SUDA and SUDC accounted for 6.10% and 0.86% of their respective mortuaries’ caseloads [11, 12]. Similarly, upon dividing the SUDY population of the present study into SUDA and SUDC groups, these groups accounted for 4.87% and 0.90% of the SRM caseload, respectively.

### Demographics and physical profile of the SUDY admissions

Males accounted for nearly two thirds of the SUDY population (62.59%), which is in agreement with local and international literature [7, 10, 12, 28]. The mean age of the SUDY population herein was similar to that found by other researchers [7, 10], although further comparison was hindered due to varying age inclusion criteria between studies. Accounting for the total SRM intake data per age category over the 10 years, toddlers (1–4 years) comprised the largest proportion of SUD admissions, suggesting that the risk for SUD is heightened during the earlier years of life (Fig. 2a).

A substantial body of data indicates that obesity and an overweight status increase the risk of cardiovascular diseases, including heart failure, coronary heart disease, and atrial fibrillation [29–31]. However, data pertaining to the association between adiposity and sudden cardiac death (SCD) is limited and inconsistent [32–37]. The significant association between being overweight and the cardiovascular system involvement in the cause of death herein suggests that being overweight is a risk factor for SCD in young people. It is of major public health importance to investigate the link between excess BMI and an increased risk for SCD in light of the increased prevalence of overweight and obesity on a global scale, as this could contribute towards the development of prevention guidelines [29]. Almirall et al. [38] identified that being underweight is a risk factor for community-acquired pneumonia which corroborates the significant association identified between the underweight status and pulmonary system herein. This finding may be explained by nutritional deficiency or underlying immune-related diseases [39].

### SUDY admission profiles

A variety of socioeconomic levels are represented in the west metropole of the City of Cape Town [40]. Within the lower socioeconomic suburbs, restricted access to medical centres and resources has been reported [41, 42]. This may explain why the low socioeconomic, densely populated neighbouring regions of Nyanga, Gugulethu and Mitchell’s Plain were distinguished SUDY hotspots in this study [43–45].

Seasonal variation in SUD has been documented in several publications suggesting that SUD may be linked to external factors such as climatic conditions [28, 46]. The seasonal peak of SUDY in winter aligns with Katz et al. [46] in the Negev Desert region of Israel. This phenomenon may be explained by the cold winter spells, which exacerbate cardiovascular and pulmonary diseases [47, 48]. Indeed, Ryti et al. [49] has identified an elevated risk of SCD with preceding cold spells.

The majority of SUDY victims being found in a ‘lying down’ position (Fig. 5a) corresponds with the literature [50, 51], however, contrast to other research, majority of our SUDY victims were found in a supine position. Majority of deaths occurred during sleep (Fig. 5c), which is consistent with majority of deaths occurring in residential settings (Fig. 5b). Indeed, there is a consistent pattern for residential locations in the literature [12, 52–54], which may indicate that most victims were unaware of a prevailing disease or condition, refused to seek or simply could not afford the necessary medical attention [53]. Therefore, initiatives should be implemented to help educate all members of society regarding the importance of recognising the warning signs and symptoms of various medical conditions, as well as the importance of seeking medical attention promptly [53].

### Cause of death and medical history

SUDs have been found to follow a similar pattern to the disease pattern prevalent in any given geographical region, and thus vary globally [53]. It is well established that the cardiovascular, pulmonary, and central nervous systems constitute the most prevalent organ systems implicated in SUD [7, 12, 53, 55], which is corroborated by this study (Table 3). Moreover, breathlessness and chest pain were two of the leading symptoms experienced prior to death (Table 5), which are cardinal symptoms of cardiovascular and pulmonary diseases [28, 56, 57].

In contrast to numerous international studies where cardiovascular disease was the leading cause of SUD [7, 10, 58], this study found that the majority of the deaths were of pulmonary origin (Table 3). This is likely related to the TB epidemic in South Africa, as South Africa is one of 30 high TB incidence countries that accounted for 87% of global estimated incidence TB cases in 2022 [59, 60].

The fact that only 48.72% cases with TB implicated in cause of death had documented prior TB-exposure during the deceased’s lifetime indicates that a large proportion of SUDY due to TB were undiagnosed prior to death. A study investigating the burden of diagnosed and undiagnosed TB in SUDC and SUDA at Tygerberg Mortuary in Cape Town found that active TB identified at autopsy was undiagnosed prior to death in an alarming 91.70% of cases [61].

The leading cause of death herein was pneumonia, accounting for 17.11% specified natural SUDY cases. This aligns with the research from developing countries [11, 62] [4, 7]. Nonetheless, multiple studies have identified pneumonia as one of the leading causes of death in non-cardiac SUD [12, 53, 55, 63]. In 2019, pneumonia and other lower respiratory tract infections were classified as the most fatal group of infectious diseases, and together ranked as the fourth cause of death globally [64]. Deaths from pneumonia comprised a larger proportion of the 1–4 year age category (25.30%) than any other age category in the present study (Table 3). It is widely known that children < 5 years of age are at greater risk for contracting pneumonia, and this may be related to their under-developed immune systems [11, 65, 66]. Indeed, in 2019, pneumonia comprised 22% of all deaths in children aged 1–5 years according to the World Health Organization, thus supporting our observation [66].

The main cause of death involving the cardiovascular system was ischaemic heart disease (Table 3), which aligns with other literature [4, 12, 63]. Although central nervous system causes of death were identified to a lower extent in the present study and appear to be less prevalent in causes of SUD in local studies [11, 12], SUD due to epilepsy (SUDEP) is an important, well-documented cause of SUD in international literature [50, 67, 68].

Communicable disease-related deaths represented at least 32.46% of SUDY, which is concerning considering communicable diseases are not only preventable but can also be treated if antibiotics are administered appropriately and timeously [69]. In the present study, post-mortem microbiology testing had only been performed in 8.60% of cases (Table 2). In light of this, it is evident that microbiological investigations should be conducted more routinely in SUD cases at post-mortem, especially when a cause of death of an infectious nature is suspected.

TB- and HIV-exposure were the two leading medical histories identified (Table 6). This is not surprising since South Africa has the world’s largest antiretroviral therapy (ART) program and has made the World Health Organization’s three global lists of high-incidence countries for TB, HIV-associated TB, and multidrug-resistant/rifampicin-resistant TB [60, 70, 71]. This may explain a considerable portion of deaths, given that HIV is known to compromise the immune system, rendering affected individuals more vulnerable to other diseases.

A positive family history was reported in 43.34% of cases, which may indicate that many of these victims failed to seek medical attention or adhere to preventative measures, or lacked the resources to do so.

Although participation in physical activity was only reported in few cases, 50% of these SUDY cases were of cardiovascular origin. This is supported by Maron et al. [72] investigating SUDY in competitive athletes in the United States. However, Corrado et al. [73] found that the increased mortality associated with physical activity in adolescents and young adults was not directly caused by the physical activity, but rather from the exercise provoking a pre-existing cardiovascular disease. Nonetheless, physical activity could not be fully investigated as the questionnaires did not specifically refer to the physical activity of the deceased, and thus, minimal cases had reported on this variable.

### Social history

Several studies have investigated and identified associations between socioeconomic status and SUD [74–76]. However, the literature is more abundant on SUD in infants (SUDI) in this regard, which hinders comparison of our study population. These studies consistently demonstrate an increased risk for SUDI with lower socioeconomic status [74, 75]. The effects of socioeconomic status are likely to be mediated by other factors, such as occupation, education, and housing, rather than being directly causal.

Health outcomes have been shown to be affected by socioeconomic status [77–80]. More specifically, individuals from lower socioeconomic backgrounds have a greater degree of risk factors for cardiovascular disease [81, 82] and demonstrate longer delays when seeking medical treatment than those from higher socioeconomic backgrounds [83]. Although the majority of SUDY victims in the present study resided in formal housing, a large proportion resided in informal housing (46.11%) (Table 4). It has been demonstrated that residents of informal settlements face a substantial health burden, as they are exposed to unsafe water, unsanitary conditions, and overcrowding, which ultimately heightens the risk for contracting infectious diseases [84–86]. This may also explain the large proportion of infectious-related deaths in our study.

Although marital status does not exclusively reflect socioeconomic status, it has been consistently associated with measures of social status, particularly in studies examining infant outcomes [75, 87, 88]. Studies have described marriage to have positive effects on the health of unemployed women by providing a source of financial and social support [24, 89]. Moreover, marriage between two working individuals implies that there are two sources of income. Thus, married individuals may be less hesitant to seek medical attention than unmarried individuals. It is therefore unsurprising that 78.03% of SUDY victims were unmarried or were minors with unmarried parents/guardians (Table 4).

Research by Chaudhari and Mohite [90] found that workers in industries, mills and shops constituted the highest number of SUD cases and attributed this to low socioeconomic status, neglect of warning signs of illness, unaffordable medical assistance, as well as physical and mental stress associated with these occupations [90]. This low affordability to medical care may be particularly relevant in the present study as 42.88% victims were unemployed (Table 4).

A key aspect of an individual’s social history is substance use. Worldwide, illicit drug use for recreational purposes remains a major problem and has been associated with a heightened risk for SUD compared with the general population [91]. In more than half the SUDY cases (54.61%), some form of substance was engaged with during the deceased’s lifetime, of which alcohol was the most prevalent (Fig. 7). Due to the ease of access to alcohol and the presence of numerous lower socioeconomic residences in the west metropole of Cape Town, individuals often have access to unlicensed alcohol-serving establishments [92]. Interestingly, underaged substance-use was noted in seven cases. This finding was unsurprising given that nationally, 49.60%, 29.50%, and 12.70% of learners had engaged with alcohol, tobacco products, and dagga in their lifetime, respectively, based on the results of a South African survey conducted in 2008 [93]. Moreover, there was a significantly higher prevalence of each of these substances in the Western Cape province when compared to the national average.

As only 32.31% of the cases had toxicology performed post-mortem (Table 2), it is likely that the prevalence of substance-use reported herein is an underestimate, thus motivating for the implementation of routine toxicology in all cases of SUD.

### Molecular autopsy candidates

Currently, molecular autopsies are being developed in a local setting to facilitate the identification of cause of death, particularly when undetermined after a thorough post-mortem investigation. Studies have documented varying resolution rates of previously unexplained SUD cases of up to 44% [94]. Thus, molecular autopsies present an appealing option for grieving relatives seeking answers [95].

The results from the molecular autopsy may bring closure to relatives and encourage them to take the necessary next steps. This may include seeking genetic counselling and testing themselves, should they be at-risk [96]. Since unresolved SUDs may have heritable origins, cardiological and genetic assessment of surviving relatives of SUD victims is recommended as this may expose a possible underlying condition and unveil presymptomatic or asymptomatic carriers [97]. The results from a molecular autopsy may also be beneficial for the family in that it may provide a more definitive family medical history and assist with family planning in cases of SUDI.

Candidates for molecular autopsies must be meticulously selected. Currently, there is a high caseload associated with forensic laboratories in South Africa which can delay ancillary investigations. Thus, molecular autopsies have the potential to alleviate this burden for both the laboratories and grieving families. For this reason, cases that are under investigation (pending ancillary testing results) are also assessed for their potential as molecular autopsy candidates. Additionally, non-specific (i.e. ‘natural causes’) and semi-specific natural deaths (i.e. ‘lung pathology’ or ‘cardiorespiratory causes’) are also assessed in the hopes of identifying a more specific cause of death, perhaps with an underlying genetic mechanism. This is important for the prevention of future SUD, particularly in genetically predisposed relatives.

The retention of biological samples at autopsy is currently at the discretion of the forensic pathologist. Although majority of the molecular autopsy candidates identified had biological samples available (90.38%) (Fig. 1), it is unfortunate that some did not. This study advocates for the mandatory retention of biological samples in undetermined cases or in ill-defined causes of death, to allow for future investigations.

### Limitations

The retrospective nature of this study meant engaging with medico-legal case files, where there is inevitable incompleteness and inherent difficulties in ensuring accuracy. Missing forms and details on forms were frequently encountered, however this could not be avoided. Several factors may have contributed to these issues, such as the extensive caseload experienced by SRM, the lengthy questionnaires, the emotional and delicate nature of interviews, the inadequate training of forensic and police officers due to resource restrictions, poor access to transportation for relatives to attend an interview at the mortuary, as well as language barriers [12]. Language barriers may be a particularly significant factor as South Africa is a highly diverse, multi-lingual country [98]. Additionally, the deceased may have been unidentified when found and thus only minimal details could be captured for these decedents. Although it is preferable to utilise more descriptive criteria when evaluating SUDY (such as time between onset of symptoms and death), the authors were limited in this regard as the National Health Act (61 of 2003) of South Africa does not specify time from symptom onset until death for inclusion in the SUD category, and this data is not always available from the case files.

### Conclusion

SUD has an important place in all medicolegal autopsies, and identifying the risk factors and lifestyle contributors to SUDY may contribute greatly to the public health sector. In addition, the diverse population of South Africa warrants investigation into the socioeconomic standing and demographics of SUDY. Being overweight was significantly associated with cardiovascular-linked SUD, while being underweight was significantly associated with pulmonary-linked SUD. Therefore, making the necessary lifestyle and dietary modifications may improve the health of those most vulnerable, and mitigate the risk for these fatalities.

A large proportion of SUDY admissions had a reported acute/chronic illness and/or family history, indicating a missed opportunity to diagnose or treat, and ultimately prevent the death. If family history of illness is present, SUDY could be prevented by early screening of risk factors and familial diseases. The leading symptoms experienced prior to death were breathlessness, vomiting (no blood), and chest pain. This finding urges public awareness, especially among healthcare workers, to recognise these early symptoms of SUD and to encourage medical intervention as soon as possible.

Furthermore, this study demonstrates the importance of post-mortem ancillary investigations in identifying cause of death in SUDY, especially in a country with a large burden of substance abuse and infectious disease. Accordingly, ancillary investigations should become routine components of post-mortem examinations of SUD. Our findings warrant that the retention of biological samples at autopsy becomes a statutory requirement in unresolved and non-specific natural SUD cases. This may assist in identifying cause of death on a molecular basis through further genetic investigations. The results of a molecular autopsy are invaluable to the surviving family as well as to the public health sectors.

## Data Availability

The data generated and/or analysed during the current study are available from the corresponding author on reasonable request.

## Declarations

### Young researcher declaration

The first author meets the inclusion criteria for the young research support criteria.

## References

[1] Dewar LJ, Krahn AD, Green MS et al (2012) 542 current practices regarding the investigation of sudden unexpected death in the young (SUDY) in Canada. Can J Cardiol 28:301–302. 10.1016/J.CJCA.2012.07.494

[2] Driscoll DJ, Edwards WD (1985) Sudden unexpected death in children and adolescents. J Am Coll Cardiol 5:118–121. 10.1016/S0735-1097(85)80540-4 3998328 10.1016/s0735-1097(85)80540-4

[3] Lim Z, Gibbs K, Potts JE, Sanatani S (2010) A review of sudden unexpected death in the young in British Columbia. Can J Cardiol 26:22–26. 10.1016/S0828-282X(10)70329-9 20101353 10.1016/s0828-282x(10)70329-9 PMC2827220

[4] Winkel BG, Holst AG, Theilade J et al (2011) Nationwide study of sudden cardiac death in persons aged 1–35 years. Eur Heart J 32:983–990. 10.1093/eurheartj/ehq428 21131293 10.1093/eurheartj/ehq428

[5] Vandayar Y, Heathfield LJ (2022) A review of the causes and risk factors for sudden unexpected death in the young. Forensic Sci Med Pathol 18:186–196. 10.1007/s12024-021-00444-3 35133622 10.1007/s12024-021-00444-3

[6] Wren C, O’Sullivan JJ, Wright C (2000) Sudden death in children and adolescents. Heart 83:410–413. 10.1136/HEART.83.4.410 10722539 10.1136/heart.83.4.410 PMC1729377

[7] Puranik R, Chow CK, Duflou JA et al (2005) Sudden death in the young. Heart Rhythm 2:1277–1282. 10.1016/j.hrthm.2005.09.008 16360077 10.1016/j.hrthm.2005.09.008

[8] Centres for Disease Control and Prevention (2016) Announcements: sudden death in the young case registry. Morb Mortal Wkly Rep 65:330–331. 10.15585/MMWR.MM6512A6 10.15585/mmwr.mm6512a6 27031082

[9] Arzamendi D, Benito B, Tizon-Marcos H et al (2011) Increase in sudden death from coronary artery disease in young adults. Am Heart J 161:574–580. 10.1016/j.ahj.2010.10.040 21392614 10.1016/j.ahj.2010.10.040

[10] Anastasakis A, Papatheodorou E, Ritsatos K et al (2018) Sudden unexplained death in the young: epidemiology, aetiology and value of the clinically guided genetic screening. Europace 20:472–480. 10.1093/EUROPACE/EUW362 28177452 10.1093/europace/euw362

[11] van Deventer BS, Rossouw SH, du Toit-Prinsloo L (2016) Sudden and unexpected childhood deaths investigated at the Pretoria Medico-Legal Laboratory, South Africa, 2007–2011. S Afr Med J 106:983–985. 10.7196/SAMJ.2016.V106I10.11028 10.7196/SAMJ.2016.v106i10.11170 27725015

[12] Tiemensma M, Burger EH (2012) Sudden and unexpected deaths in an adult population, Cape Town, South Africa, 2001–2005. S Afr Med J 102:90–94. 10.7196/samj.5363 22310440 10.7196/samj.5363

[13] Corrado D, Basso C, Thiene G (2001) Sudden cardiac death in young people with apparently normal heart. Cardiovasc Res 50:399–408. 10.1016/s0008-6363(01)00254-1 11334844 10.1016/s0008-6363(01)00254-1

[14] Eckart RE, Scoville SL, Campbell CL et al (2004) Sudden death in young adults: a 25-year review of autopsies in military recruits. Ann Intern Med 141:829–834. 10.7326/0003-4819-141-11-200412070-00005 15583223 10.7326/0003-4819-141-11-200412070-00005

[15] Boczek NJ, Tester DJ, Ackerman MJ (2012) The molecular autopsy: an indispensable step following sudden cardiac death in the young? Herzschr Elektrophys 23:167–173. 10.1007/S00399-012-0222-X 10.1007/s00399-012-0222-x PMC3852896 22993115

[16] Sanchez O, Campuzano O, Fernández-Falgueras A et al (2016) Natural and undetermined sudden death: value of post-mortem genetic investigation. PLoS ONE 11:e0167358. 10.1371/journal.pone.0167358 27930701 10.1371/journal.pone.0167358 PMC5145162

[17] Tester DJ, Medeiros-Domingo A, Will ML et al (2012) Cardiac channel molecular autopsy: insights from 173 consecutive cases of autopsy-negative sudden unexplained death referred for postmortem genetic testing. Mayo Clin Proc 87:524–539. 10.1016/J.MAYOCP.2012.02.017 22677073 10.1016/j.mayocp.2012.02.017 PMC3498431

[18] Tester DJ, Ackerman MJ (2007) Postmortem long QT syndrome genetic testing for sudden unexplained death in the young. J Am Coll Cardiol 49:240–246. 10.1016/J.JACC.2006.10.010 17222736 10.1016/j.jacc.2006.10.010

[19] Bates K, Sweeting J, Yeates L et al (2019) Psychological adaptation to molecular autopsy findings following sudden cardiac death in the young. Genet Med 21:1452–1456. 10.1038/S41436-018-0338-4 30327538 10.1038/s41436-018-0338-4

[20] Reid KM, Martin LJ, Heathfield LJ (2020) Bodies without names: a retrospective review of unidentified decedents at Salt River Mortuary, Cape Town, South Africa, 2010–2017. S Afr Med J 110:223–228. 10.7196/SAMJ.2020.V110I3.14192 32657700 10.7196/SAMJ.2020.v110i3.14192

[21] World Health Organization (2007) BMI-for-age (5–19 years). https://www.who.int/tools/growth-reference-data-for-5to19-years/indicators/bmi-for-age. Accessed 27 Oct 2022

[22] World Health Organization (n d.) Body mass index-for-age (BMI-for-age). https://www.who.int/toolkits/child-growth-standards/standards/body-mass-index-for-age-bmi-for-age. Accessed 27 Oct 2022

[23] Hu Y, Goldman N (1990) Mortality differentials by marital status: an international comparison. Demography 27:233–250. 10.2307/2061451 2332088

[24] Wyke S, Ford G (1992) Competing explanations for associations between marital status and health. Soc Sci Med 34:523–532. 10.1016/0277-9536(92)90208-8 1604359 10.1016/0277-9536(92)90208-8

[25] South African Government (1959) The Inquests Act 58 of 1959. https://www.gov.za/sites/default/files/gcis_document/201505/act-58-1959.pdf. Accessed 24 August 2022

[26] Doolan A, Langlois N, Semsarian C (2004) Causes of sudden cardiac death in young australians. Med J Aust 180:110–112. 10.5694/j.1326-5377.2004.tb05830.x 14748671 10.5694/j.1326-5377.2004.tb05830.x

[27] Mole CG (2019) Trends in manner and cause of death over a 10-year period (2007–2016) at a regional forensic pathology facility, Cape Town, South Africa. Aust J Forensic Sci 51:197–200. 10.1080/00450618.2019.1569134

[28] Nofal HK, Abdulmohsen MF, Khamis AH (2011) Incidence and causes of sudden death in a university hospital in Eastern Saudi Arabia. East Mediterr Health J 17:665–670. 10.26719/2011.17.9.665 22259916

[29] Aune D, Schlesinger S, Norat T, Riboli E (2018) Body mass index, abdominal fatness, and the risk of sudden cardiac death: a systematic review and dose–response meta-analysis of prospective studies. Eur J Epidemiol 33:711–722. 10.1007/S10654-017-0353-9 29417316 10.1007/s10654-017-0353-9 PMC6061127

[30] Aune D, Sen A, Norat T et al (2016) Body mass index, abdominal fatness, and heart failure incidence and mortality: a systematic review and dose-response meta-analysis of prospective studies. Circulation 133:639–649. 10.1161/CIRCULATIONAHA.115.016801 26746176 10.1161/CIRCULATIONAHA.115.016801

[31] de Rosa R, Vasa-Nicotera M, Leistner DM et al (2017) Coronary atherosclerotic plaque characteristics and cardiovascular risk factors - insights from an optical coherence tomography study. Circ J 81:1165–1173. 10.1253/CIRCJ.CJ-17-0054 28420816 10.1253/circj.CJ-17-0054

[32] Chiuve SE, Sun Q, Sandhu RK et al (2015) Adiposity throughout adulthood and risk of sudden cardiac death in women. JACC Clin Electrophysiol 1:520–528. 10.1016/J.JACEP.2015.07.011 26824079 10.1016/j.jacep.2015.07.011 PMC4725590

[33] Eranti A, Aro AL, Kerola T et al (2016) Body mass index as a predictor of sudden cardiac death and usefulness of the electrocardiogram for risk stratification. Am J Cardiol 117:388–393. 10.1016/J.AMJCARD.2015.10.057 26723105 10.1016/j.amjcard.2015.10.057

[34] Jouven X, Desnos M, Guerot C, Ducimetière P (1999) Predicting sudden death in the population: the Paris prospective study I. Circulation 99:1978–1983. 10.1161/01.CIR.99.15.1978 10209001 10.1161/01.cir.99.15.1978

[35] Wannamethee G, Shaper AG, Macfarlane PW, Walker M (1995) Risk factors for sudden cardiac death in middle-aged British men. Circulation 91:1749–1756. 10.1161/01.CIR.91.6.1749 7882483 10.1161/01.cir.91.6.1749

[36] Ohira T, Maruyama M, Imano H et al (2012) Risk factors for sudden cardiac death among Japanese: the circulatory risk in communities study. J Hypertens 30:1137–1143. 10.1097/HJH.0B013E328352AC16 22573081 10.1097/HJH.0b013e328352ac16

[37] Chei CL, Iso H, Yamagishi K et al (2007) Body mass index and weight change since 20 years of age and risk of coronary heart disease among Japanese: the Japan public health center-based study. Int J Obes 32:144–151. 10.1038/sj.ijo.0803686 10.1038/sj.ijo.0803686 17637701

[38] Almirall J, Bolíbar I, Serra-Prat M et al (2008) New evidence of risk factors for community-acquired pneumonia: a population-based study. Eur Respir J 31:1274–1284. 10.1183/09031936.00095807 18216057 10.1183/09031936.00095807

[39] Hedlund J, Hansson LO, Örtqvist Å (1995) Short- and long-term prognosis for middle-aged and elderly patients hospitalized with community-acquired pneumonia: impact of nutritional and inflammatory factors. Scand J Infect Dis 27:32–37. 10.3109/00365549509018970 7540316 10.3109/00365549509018970

[40] Clark C, Mole CG, Heyns M (2017) Patterns of blunt force homicide in the West Metropole of the City of Cape Town, South Africa. S Afr J Sci 113:1–6. 10.17159/sajs.2017/20160214

[41] Taani I, Muller G (2012) Violence prevention through urban upgrading: Nyanga-Gugulethu baseline survey. https://www.datafirst.uct.ac.za/dataportal/index.php/catalog/473/download/6221. Accessed 12 October 2022

[42] Lambert RF, Yu A, Orrell C, Haberer JE (2020) Perceived oral health interventions by medical providers in Gugulethu, South Africa. PLoS ONE 15:e0233437. 10.1371/journal.pone.0233437 32453785 10.1371/journal.pone.0233437 PMC7250410

[43] Mitchell’s Plain (Main Place (2024) City of Cape Town, South Africa) - Population Statistics, Charts, Map and Location. https://www.citypopulation.de/en/southafrica/cityofcapetown/199039__mitchells_plain/. Accessed 10

[44] Nyanga (Main Place (2024) City of Cape Town, South Africa) - Population Statistics, Charts, Map and Location. https://www.citypopulation.de/en/southafrica/cityofcapetown/199031__nyanga/. Accessed 10

[45] Gugulethu (Main Place (2024) City of Cape Town, South Africa) - Population Statistics, Charts, Map and Location. https://www.citypopulation.de/en/southafrica/cityofcapetown/199030__gugulethu/. Accessed 10

[46] Katz A, Biron A, Ovsyshcher E, Porath A (2000) Seasonal variation in sudden death in the Negev desert region of Israel. Isr Med Assoc J 2:17–21 10892365

[47] Ayres JG (1986) Seasonal pattern of acute bronchitis in general practice in the United Kingdom 1976-83. Thorax 41:106–110. 10.1136/THX.41.2.106 3704975 10.1136/thx.41.2.106 PMC460271

[48] Fares A (2013) Winter cardiovascular diseases phenomenon. N Am J Med Sci 5:266–279. 10.4103/1947-2714.110430 23724401 10.4103/1947-2714.110430 PMC3662093

[49] Ryti NRI, Mäkikyrö EMS, Antikainen H et al (2017) Risk of sudden cardiac death in relation to season-specific cold spells: a case–crossover study in Finland. BMJ Open 7:e017398. 10.1136/BMJOPEN-2017-017398 29127226 10.1136/bmjopen-2017-017398 PMC5695410

[50] Kloster R, Engelskjøn T (1999) Sudden unexpected death in epilepsy (SUDEP): a clinical perspective and a search for risk factors. J Neurol Neurosurg Psychiatry 67:439–444. 10.1136/JNNP.67.4.439 10486388 10.1136/jnnp.67.4.439 PMC1736592

[51] Hesdorffer DC, Crandall LA, Friedman D, Devinsky O (2015) Sudden unexplained death in childhood: a comparison of cases with and without a febrile seizure history. Epilepsia 56:1294–1300. 10.1111/EPI.13066 26120007 10.1111/epi.13066

[52] Romero JL, Gálvez JLL, Gonzalez LR (2017) Pulmonary embolism as a cause of unexpected sudden death in people aged 1–35 years. Cuban Soc Cardiol 9:215–217

[53] Pelemo OE, Sabageh D, Komolafe AO et al (2014) An autopsy review of sudden unexpected natural deaths in a suburban Nigerian population. Popul Health Metr 12:1–6. 10.1186/s12963-014-0026-924479861

[54] Risgaard B, Winkel BG, Jabbari R et al (2014) Burden of sudden cardiac death in persons aged 1 to 49 years nationwide study in Denmark. Circ Arrhythm Electrophysiol 7:205–211. 10.1161/CIRCEP.113.001421 24604905 10.1161/CIRCEP.113.001421

[55] Kumar V, San KP, Idwan A et al (2007) A study of sudden natural deaths in medico legal autopsies in University Malaya Medical Centre (UMMC), Kuala Lumpur. J Forensic Leg Med 14:151–154. 10.1016/J.JCFM.2006.05.005 16914354 10.1016/j.jcfm.2006.05.005

[56] Smoller JW, Pollack MH, Otto MW et al (1996) Panic anxiety, dyspnea, and respiratory disease. Theoretical and clinical considerations. Am J Respir Crit Care Med 154:6–17. 10.1164/AJRCCM.154.1.8680700 8680700 10.1164/ajrccm.154.1.8680700

[57] Jany B (2017) Pulmonary causes of chest pain. Internist 58:22–28. 10.1007/S00108-016-0169-9 27986981 10.1007/s00108-016-0169-9

[58] Drory Y, Turetz Y, Hiss Y et al (1991) Sudden unexpected death in persons < 40 years of age. Am J Cardiol 68:1388–1392. 10.1016/0002-9149(91)90251-F 1951130 10.1016/0002-9149(91)90251-f

[59] van der Walt M, Moyo S, Pillay Y et al (2021) The first national TB prevalence survey: South Africa 2018. https://www.nicd.ac.za/wp-content/uploads/2021/02/TB-Prevalence-survey-report_A4_SA_TPS-Short_Feb-2021.pdf. Accessed 18 Oct 2022

[60] World Health Organization (2023) Global tuberculosis report 2023. https://iris.who.int/bitstream/handle/10665/373828/9789240083851-eng.pdf?sequence=1. Accessed 16 Jan 2024

[61] Osman M, Verster J, Dempers JJ et al (2021) Tuberculosis in persons with sudden unexpected death, in Cape Town, South Africa. Int J Infect Dis 105:75–82. 10.1016/J.IJID.2021.02.036 33582368 10.1016/j.ijid.2021.02.036 PMC8358423

[62] Mohamed EY, Abdelbadie A, Abdalla SM et al (2008) Sudden natural death in Khartoum Mortuary. Sud J Med Sc 3:319–324. 10.4314/sjms.v3i4.38553

[63] Daş T, Buğra A (2022) Natural causes of sudden young adult deaths in forensic autopsies. Cureus 14:2–6. 10.7759/CUREUS.21856 10.7759/cureus.21856 PMC8901078 35273838

[64] World Health Organization (2020) WHO reveals leading causes of death and disability worldwide: 2000–2019. https://www.who.int/news/item/09-12-2020-who-reveals-leading-causes-of-death-and-disability-worldwide-2000-2019. Accessed 19 Oct 2022

[65] World Health Organization (2021) Pneumonia. https://www.who.int/news-room/fact-sheets/detail/pneumonia. Accessed 19 Oct 2022

[66] World Health Organization (2022) Pneumonia in children. https://www.who.int/news-room/fact-sheets/detail/pneumonia. Accessed 11 Jan 2024

[67] Langan Y, Nashef L, Sander JWAS (2000) Sudden unexpected death in epilepsy: a series of witnessed deaths. J Neurol Neurosurg Psychiatry 68:211–213. 10.1136/JNNP.68.2.211 10644790 10.1136/jnnp.68.2.211 PMC1736784

[68] Hitiris N, Suratman S, Kelly K et al (2007) Sudden unexpected death in epilepsy: a search for risk factors. Epilepsy Behav 10:138–141. 10.1016/J.YEBEH.2006.11.010 17196884 10.1016/j.yebeh.2006.11.010

[69] Drexler M, Institute of Medicine (US) (2010) IV Prevention and treatment. What you need to know about infectious disease. National Academies Press (US), Washington DC 24983051

[70] UNAIDS (2017) Country factsheets South Africa. http://www.unaids.org/en/regionscountries/countries/southafrica. Accessed 15 October 2022

[71] Preface National Strategic Plan for HIV, TB and STIs (2028) 2023– https://sanac.org.za/wp-content/uploads/2023/05/NSP-Document.pdf. Accessed 16 Jan 2024

[72] Maron BJ, Doerer JJ, Haas TS et al (2009) Sudden deaths in young competitive athletes. Circulation 119:1085–1092. 10.1161/CIRCULATIONAHA.108.804617 19221222 10.1161/CIRCULATIONAHA.108.804617

[73] Corrado D, Basso C, Rizzoli G et al (2003) Does sports activity enhance the risk of sudden death in adolescents and young adults? J Am Coll Cardiol 42:1959–1963. 10.1016/J.JACC.2003.03.002 14662259 10.1016/j.jacc.2003.03.002

[74] Valdés-Dapena M, Birle LJ, McGovern JA et al (1968) Sudden unexpected death in infancy: a statistical analysis of certain socioeconomic factors. J Pediatr 73:387–394. 10.1016/S0022-3476(68)80116-7 5667421

[75] Spencer N, Logan S (2004) Sudden unexpected death in infancy and socioeconomic status: a systematic review. J Epidemiol Community Health 58:366–373. 10.1136/JECH.2003.011551 15082732 10.1136/jech.2003.011551 PMC1732769

[76] Warming PE, Ågesen FN, Lynge TH et al (2023) The impact of modifiable risk factors in the association between socioeconomic status and sudden cardiac death in a prospective cohort study: equal access to healthcare, unequal outcome. Eur J Prev Cardiol 30:1526–1534. 10.1093/EURJPC/ZWAD086 36943322 10.1093/eurjpc/zwad086

[77] Holme I, Helgeland A, Hjermann I et al (1980) Four-year mortality by some socioeconomic indicators: the Oslo study. J Epidemiol Community Health 34:48–52. 10.1136/jech.34.1.48 7365395 10.1136/jech.34.1.48 PMC1052040

[78] Kaplan GA, Keil JE (1993) Socioeconomic factors and cardiovascular disease: a review of the literature. Circulation 88:1973–1998. 10.1161/01.CIR.88.4.1973 8403348 10.1161/01.cir.88.4.1973

[79] Loucks EB, Lynch JW, Pilote L et al (2009) Life-course socioeconomic position and incidence of coronary heart disease: the Framingham offspring study. Am J Epidemiol 169:829–836. 10.1093/AJE/KWN403 19179358 10.1093/aje/kwn403 PMC2727217

[80] Frijters P, Haisken-DeNew JP, Shields MA (2005) The causal effect of income on health: evidence from German reunification. J Health Econ 24:997–1017. 10.1016/J.JHEALECO.2005.01.004 16129130 10.1016/j.jhealeco.2005.01.004

[81] Choinière R, Lafontaine P, Edwards AC (2000) Distribution of cardiovascular disease risk factors by socioeconomic status among Canadian adults. Can Med Assoc J 162:S13–S24 10813023 PMC1232440

[82] Davari M, Maracy MR, Khorasani E (2019) Socioeconomic status, cardiac risk factors, and cardiovascular disease: a novel approach to determination of this association. ARYA Atheroscler 15:260–266. 10.22122/ARYA.V15I6.1595 32206069 10.22122/arya.v15i6.1595 PMC7073799

[83] Foraker RE, Rose KM, McGinn AP et al (2008) Neighborhood income, health insurance, and prehospital delay for myocardial infarction: the atherosclerosis risk in communities study. Arch Intern Med 168:1874–1879. 10.1001/ARCHINTE.168.17.1874 18809814 10.1001/archinte.168.17.1874 PMC4682553

[84] Ezeh A, Oyebode O, Satterthwaite D et al (2017) The history, geography, and sociology of slums and the health problems of people who live in slums. Lancet 389:547–558. 10.1016/S0140-6736(16)31650-6 27760703 10.1016/S0140-6736(16)31650-6

[85] World Health Organization and UN-Habitat (2016) Global report on urban health: equitable, healthier cities for sustainable development. https://www.who.int/publications/i/item/9789241565271. Accessed 13 Jan 2024

[86] World Health Organization (2023) Sanitation. https://www.who.int/news-room/fact-sheets/detail/sanitation. Accessed 13 Jan 2024

[87] Winterbach M, Hattingh C, Heathfield LJ (2021) Retrospective study of sudden unexpected death of infants in the garden route and central karoo districts of South Africa: causes of death and epidemiological factors. S Afr J Child Health 15:74–82. 10.7196/SAJCH.2021.V15.I2.1729

[88] Radojevic N, Konatar J, Vukcevic B et al (2021) The socio-economic status of families experiencing the sudden unexpected death of an infant – is it possibly related to a higher rate of non-natural deaths among them. J Forensic Leg Med 80:102168. 10.1016/j.jflm.2021.102168 33878589 10.1016/j.jflm.2021.102168

[89] Waldron I, Hughes ME, Brooks TL (1996) Marriage protection and marriage selection-prospective evidence for reciprocal effects of marital status and health. Soc Sci Med 43:113–123. 10.1016/0277-9536(95)00347-9 8816016 10.1016/0277-9536(95)00347-9

[90] Chaudhari V, Mohite S (2012) Current trends in sudden natural deaths. J Forensic Med Sci Law 21:1–8

[91] Fischbach P (2017) The role of illicit drug use in sudden death in the young. Cardiol Young 27:S75–S79. 10.1017/S1047951116002274 28084963 10.1017/S1047951116002274

[92] Towards a safer Khayelitsha (2014) Report of the commission of inquiry into allegations of police inefficiency and a breakdown in relations between SAPS and the community of Khayelitsha. https://www.westerncape.gov.za/files/khayelitsha_commission_report.pdf. Accessed 14 Jan 2024

[93] Reddy S, James S, Sewpaul R et al (2008) Umthente Uhlaba Usamila - The 2nd South African national youth risk behaviour survey. https://granthaskin.files.wordpress.com/2012/06/youth-risk-behaviour-survey-2008_final_report.pdf. Accessed 19 Oct 2022

[94] Anderson JH, Tester DJ, Will ML, Ackerman MJ (2016) Whole-exome molecular autopsy after exertion-related sudden unexplained death in the young. Circ Cardiovasc Genet 9:259–265. 10.1161/CIRCGENETICS.115.001370/-/DC1 27114410 10.1161/CIRCGENETICS.115.001370

[95] Heathfield LJ, Martin LJ, Ramesar R (2019) Consideration of molecular autopsies in forensic cases of sudden unexpected death in infants and children in South Africa. S Afr J Child Health 13:2–3. 10.7196/SAJCH.2019.v13i1.1573

[96] Burns KM, Bienemann L, Camperlengo L et al (2017) The sudden death in the young case registry: collaborating to understand and reduce mortality. J Pediatr 139:e20162757. 10.1542/peds.2016-2757 10.1542/peds.2016-2757 PMC5330401 28228502

[97] Tan HL, Hofman N, Van Langen IM et al (2005) Sudden unexplained death. Circulation 112:207–213. 10.1161/CIRCULATIONAHA.104.522581 15998675 10.1161/CIRCULATIONAHA.104.522581

[98] Statistics South Africa (2012) South African Statistics 2012. https://www.statssa.gov.za/publications/SAStatistics/SAStatistics2012.pdf. Accessed 29 Oct 2022

